# Predicting Postpartum Depressive Symptoms from Pregnancy Biopsychosocial Factors: A Longitudinal Investigation Using Structural Equation Modeling

**DOI:** 10.3390/ijerph17228445

**Published:** 2020-11-14

**Authors:** Verónica Martínez-Borba, Carlos Suso-Ribera, Jorge Osma, Laura Andreu-Pejó

**Affiliations:** 1 Department of Basic and Clinical Psychology and Psychobiology, Jaume I University, Avda, Vicent Sos Baynat s/n, 12071 Castellon de la Plana, Spain; borba@uji.es (V.M.-B.); susor@uji.es (C.S.-R.); 2 Instituto de Investigación Sanitaria de Aragón, C/ San Juan Bosco, 13, 50009 Zaragoza, Spain; pejo@uji.es; 3 Department of Psychology and Sociology, Universidad de Zaragoza, C/ Atarazanas, 4, 44003 Teruel, Spain; 4 Nursing Department, Jaume I University, Avda, Vicent Sos Baynat s/n, 12071 Castellon de la Plana, Spain

**Keywords:** pregnancy, postpartum, depressive symptoms, risk factors, biopsychosocial, longitudinal studies, information and communication technologies

## Abstract

The prediction of postpartum depression (PPD) should be conceptualized from a biopsychosocial perspective. This study aims at exploring the longitudinal contribution of a set of biopsychosocial factors for PPD in perinatal women. A longitudinal study was conducted, assessment was made with a website and included biopsychosocial factors that were measured during pregnancy (n = 266, weeks 16–36), including age, affective ambivalence, personality characteristics, social support and depression. Depression was measured again at postpartum (n = 101, weeks 2–4). The analyses included bivariate associations and structural equation modeling (SEM). Age, affective ambivalence, neuroticism, positive, and negative affect at pregnancy were associated with concurrent depression during pregnancy (all *p* < 0.01). Age, affective ambivalence, positive affect, and depression at pregnancy correlated with PPD (all *p* < 0.05). Affective ambivalence (β = 1.97; *p* = 0.003) and positive (β = −0.29; *p* < 0.001) and negative affect (β = 0.22; *p* = 0.024) at pregnancy remained significant predictors of concurrent depression in the SEM, whereas only age (β = 0.27; *p* = 0.010) and depression (β = 0.37; *p* = 0.002) at pregnancy predicted PPD. Biopsychosocial factors are clearly associated with concurrent depression at pregnancy, but the stability of depression across time limits the prospective contribution of biopsychosocial factors. Depression should be screened early during pregnancy, as this is likely to persist after birth. The use of technology, as in the present investigation, might be a cost-effective option for this purpose.

## 1. Introduction

Postpartum depression (PPD) is one of the most prevalent emotional disorders worldwide, with global estimates ranging from between 5% to 25% [1]. PPD is associated with both personal and economic burden [2]. First, negative health consequences for the women and the baby have been reported, such as inadequate gestational weight gain, low birth weight, preterm birth, inadequate weight gain in the baby, under-utilization of prenatal care services, increased substance use in the women, and increased maternal mortality [3,4]. Second, the economic costs of PPD include loss of productivity and health care expenses [5]. Not surprisingly, the American Psychiatric Association has indicated that all perinatal women should be assessed for both the presence of and risk for psychiatric disorders during pregnancy (twice), and during the first six months postpartum [6].

The biopsychosocial model of PPD is an attempt to comprehensively understand the risk for the onset of depressive symptoms, but also the protective factors associated with this disorder, so that prevention and treatment efforts can be developed in a more effective manner [7]. Several meta-analyses and systematic reviews have supported this biopsychosocial approach to PPD [8,9]. As a result of this, factors such as low educational and income level, ambivalence toward pregnancy, personal and family history of depression, perceived social support, and personality characteristics, among others, are now frequently investigated in the PPD literature [8,9]. Specifically, variables related to the increased risk of PPD are pregnancy ambivalence, neuroticism, negative affect and prenatal depressive symptoms [10,11,12]. On the contrary, positive affect and extraversion seem to be protective factors for PPD development [11,12]. There is sufficient evidence, however, that the best predictor of PPD is depressive symptomatology during pregnancy [8,9,13].

The research to date has clearly contributed to the development of the biopsychosocial approach to PPD. Nevertheless, there are a number of shortcomings in the literature on PPD that are yet to be addressed. For instance, most studies tend to investigate the contribution of only one or two risk factors altogether (e.g., Hetherington, McDonald, Williamson, Patten and Tough, 2018 [14]), so the communalities between biopsychosocial factors are not controlled and the unique contribution of each variable remains unclear [15]. This is important as it might help reduce the number of therapeutic targets to a more manageable set. Additionally, studies exploring the relationship between biopsychosocial factors and perinatal depressive symptoms are generally cross-sectional (e.g., Adamu and Adinew, 2018 [16]), so the predictive value of these variables in the evolution of depressive symptoms across the perinatal period is unclear. Research has shown that both the spontaneous remission and intensification of depressive symptoms exist [17], so longitudinal studies are fundamental if prevention and treatment programs are to be effectively developed.

In addition to the aforementioned shortcomings, previous research has revealed the important barriers to face-to-face evaluation in the perinatal period, including insufficient time for care providers, availability of mental health services, lack of time for the women, difficulties in combining child care with onsite appointments, geographical distance, and the high economic costs of traveling to academic or health care institutions [18]. The use of information and communication technologies (ICT) in the field of health (e-health) has emerged as an alternative to traditional face-to-face methods in response to these barriers. To the best of our knowledge, there is only one longitudinal study that used ICTs (e-mail) to explore emotional disorders in perinatal women [19]. However, a biopsychosocial approach to PPD was not adopted in this investigation, and complex associations between variables, for example via structural equation modeling (SEM), were not investigated.

With the aim of providing more robust evidence for the development of screening, prevention, and treatment programs for PPD, we have conducted a study exploring the unique contribution of a set of risk and protective biopsychosocial factors for PPD in perinatal women using SEM. A longitudinal design was implemented, and prenatal and postpartum assessments were conducted using ICT (i.e., a website). After a thorough literature research, the biopsychosocial factors included in the prediction of PPD were psychosocial factors with robust support in the literature (ambivalence toward pregnancy, neuroticism, extraversion, positive and negative affect, social support, and prenatal depressive symptoms) and a biological variable, namely age, with limited but promising evidence [8].

On the basis of previous research, we hypothesize that the perinatal risk factors for concurrent and prospective PPD will include age (i.e., being younger), affective ambivalence, neuroticism, and negative affect. On the contrary, we expect that extraversion, positive affect and social support will be protective factors for depressive symptoms. We anticipate that prenatal depressive symptoms will be the best predictors of PPD. We also expect that psychosocial factors will be intercorrelated, so that only a small subset of them will uniquely contribute to PPD in the SEM. The unique contribution of these variables is difficult to anticipate from the existing literature, and will be investigated in an exploratory manner. By testing these hypotheses, we expect to achieve our study goal, that is, explore the unique prospective contribution of a set of biopsychosocial factors in the mother in the prediction of PDD. To make the text more readable, we will interchangeably use the terms pregnancy and prenatal when referring to the assessments made before delivery. 

## 2. Materials and Methods 

### 2.1. Participants

**Figure 1:**
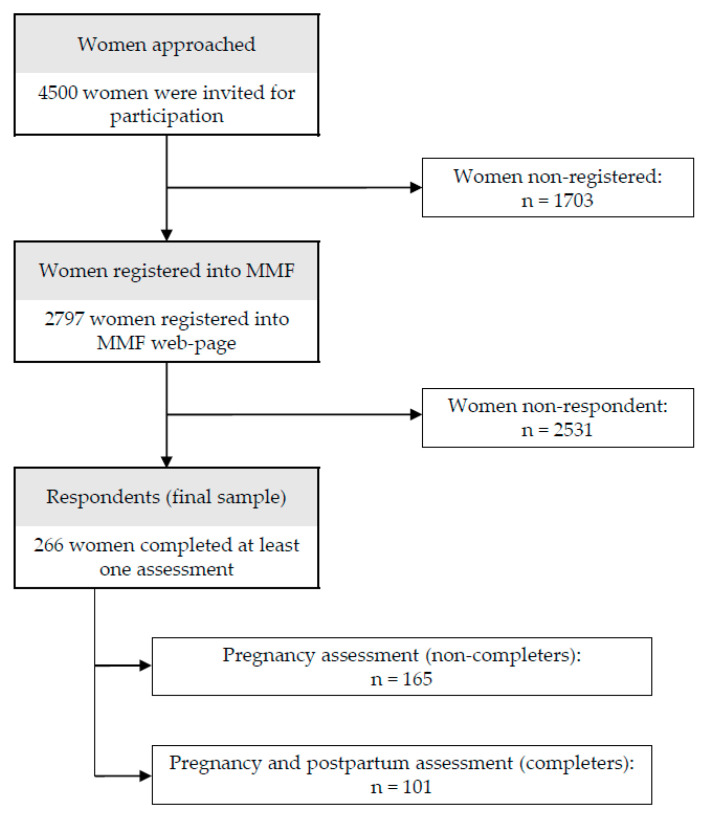
Flow diagram of participants.

The study was conducted between 2012 and 2015. The sample included 266 women who voluntarily registered on the MamáFeliz (HappyMom, hereafter MMF) website for assessment and completed the first evaluation (during pregnancy). These women were asked to respond to two assessments: one during pregnancy (between week 16 and week 36) and one at postpartum (between weeks 2 and 4 after delivery, according to the women’s availability). During pregnancy, depression has been shown to be more prevalent during the second and the third trimesters [20]. At postpartum, the highest prevalence rates of depression were found between weeks 2 and 7 after delivery [21,22]. As observed in Figure 1, from the initial sample, 101 women also provided information in the postpartum (completers’ sample). Sociodemographic characteristics of the sample, divided by group (completers vs. non-completers), will be described in the results section. Several guidelines have been proposed in the multiple regression literature for deciding the sample size required for analyses. For example, Harrell (2001) proposed a minimum of 10 participants for each predictor in the model. Our SEM included 8 predictors, and therefore we would require 80 participants according to this rule of thumb. Harris (2001) proposed at least n = 50 + *p*, where n is sample size and *p* is number of predictors. According to this, the minimum n would be 58 participants. Based on previous recommendations on required sample sizes when conducting multiple regressions [23,24], the current sample size was sufficient for the analyses conducted.

### 2.2. Procedure

All the procedures described in the present study were approved by the ethical committees of the Hospital Universitario La Plana de Villareal (Castellón) and the Gobierno de Aragón (CP12/2012). The health professionals at the specialized public gynecology centers collaborating in the study disseminated the investigation with all consecutive potential participants meeting the eligibility criteria (see Table 1) and gave women a document with a unique code and the link to the MMF website. Once on the website, the women that voluntarily agreed to participate in the study had to accept the data protection and confidentiality policies and had to sign the online informed consent form before completing the online assessments. Health professionals participating in the study gave the information on how to participate to pregnant women meeting the inclusion criteria. However, the number of women not meeting the criteria or not willing to participate was not reliably collected by the health care providers due to time restrictions during consultations.

The assessments were carried out entirely via the MMF website, which evaluated the most important biopsychosocial factors associated with perinatal depressive symptoms. E-mail reminders were used for the prospective assessments. At the end of each assessment, women received feedback about their present mental well-being as a result of their evaluation. If depressive symptoms were detected after the assessments, they received an e-mail recommending a consultation with their doctor for a more in-depth evaluation of their emotional state.

### 2.3. Measures

All sociodemographic and biopsychosocial data were assessed online via the MMF website. All the questionnaires mentioned below were administered in their full length. 

#### 2.3.1. Demographic and Biologic

We asked for nationality, educational level, relationship status and age; ad hoc questions were developed. To report on their nationality, women had to indicate the country (Spain only) or the region (i.e., South America or Central America, North America, Western Europe, Eastern Europe, North Africa, South Africa, Middle East countries, Far East countries, Southeast Asia, and Oceania) they were born in. Regarding educational level, women responded as to whether they had no studies, basic studies, secondary studies, bachelor studies, technical studies, university studies, a master’s degree or doctorate studies. Women also indicated their marital status as follows: single, unmarried (with a stable partner), married, divorced, or widowed.

#### 2.3.2. Psychological

Ambivalence toward pregnancy: This ad hoc item evaluated mixed feelings about pregnancy; “How often have you experienced feelings of ambivalence and/or contradiction about your pregnancy (for example, I have sometimes experienced joy, but also sadness or hope, but also worry)”. This item has 4 response options ranging from 0 = “never” to 3 = “very often”. Higher scores represent more ambivalence toward pregnancy.

The Beck Depression Inventory (BDI-II) [25,26]: This comprises 21 items and evaluates depressive symptoms and cognitions experienced during the last two weeks. Each item has 4 response options (0 = “not at all” and 3 = “severely”), so the total score can range from 0 to 63. Higher scores indicate more severe depressive symptomatology. The recommended BDI–II cutoffs for women in Spain are 0–13 for minimal depressive symptoms, 14–19 for mild depressive symptoms, 20–28 for moderate depressive symptoms, and 29–63 for severe depressive symptoms [25,27]. In our sample, the reliability was good during both pregnancy (α = 0.81) and postpartum (α = 0.85).

The Positive and Negative Affect Schedule (PANAS) [28,29]: This evaluates positive and negative emotional experiences. It consists of 20 adjectives, 10 for each affect scale (i.e., Positive and Negative). Each item is divided into 5 grades (1 = “very slightly/not at all” and 5 = “extremely”) and total scores for each scale can range from 10 to 50. In our sample, Cronbach alphas were very good (0.89 and 0.86 for positive and negative affect, respectively).

The Eysenck Personality Questionnaire revised (EPQ-R) [30,31]: This assesses patterns of behaviors, thoughts, and feelings (i.e., personality). The questionnaire is composed of 48 dichotomous (Yes/No) items (12 for extraversion, 12 for neuroticism, 12 for psychoticism, and 12 for lie). For this study we only used the neuroticism (α = 0.79) and extraversion (α = 0.77) scales. 

#### 2.3.3. Social

The Multidimensional Scale of Perceived Social Support (MSPSS) [32,33]: This is composed of 12 items that evaluate perceived social support in three domains (family, friends, and others). Each item has 7 response options (1 = “completely disagree” and 7 = “completely agree”). Total scores have a 12–84 range. Higher scores represent a higher perception of social support. In our sample, the psychometric properties of the three subscales were very good (0.91 ≤ α ≤ 0.94), but the total MSPSS score was preferred to reduce the number of statistical comparisons between biopsychosocial variables included in this study and to reduce the risk of type I errors.

### 2.4. Data Analysis

First, we conducted a descriptive analysis comparing completers and non-completers using a Chi-squared test for categorical variables and a Mann–Whitney *U* test for continuous factors (a non-parametric test was selected due to the distribution of scores). This comparison between completers and non-completers was made to evaluate whether there were baseline differences between both samples that would help us understand dropouts and therefore would compromise the generalizability of the findings. This comparison, however, is not related to the main goal of the present work (i.e., to predict postpartum depressive symptoms from a biopsychosocial perspective). For the same reason, we calculated Spearman and not Pearson correlations to explore the relationship between prenatal psychosocial factors (age, affective ambivalence, personality, affect, and social support) and prenatal and PPD, considering the completers’ sample only. Finally, SEM analyses were conducted to test a model that accounts for PPD from prenatal characteristics in the sample of completers. SEM has become a technique of choice when exploring complex associations between variables because of its advantages [34]. To name some examples, SEM allows the exploration of several relationships at the same time and facilitates the simultaneous use of a construct both as a dependent (i.e., prenatal depressive symptoms predicted by prenatal psychosocial factors) and as an independent variable (i.e., prenatal depressive symptoms predicting PPD) [35]. The fit for the proposed model was assessed with the Chi-square test, the root mean square error of approximation (RMSEA), the standardized root mean square residual (SRMR), the Tucker–Lewis index (TLI), and the comparative fit index (CFI). RMSEA and SRMR values smaller than 0.05 and TLI and CFI values greater than 0.95 reflect an excellent fit [36]. As recommended in the literature [37], maximum likelihood parameter estimation with standard errors and a mean-adjusted Chi-square test that is robust to non-normality (Satorra–Bentler MLM estimation) was used in the SEM. 

Past research has indicated that adjustment to the alpha level is not necessary when exploratory studies include simple rather than intricate sets of hypotheses [38]. The present study’s hypothesis is simple in the sense that we only anticipate that a reduced number of predictors will emerge from the SEM, while the exact unique contributions and their directions are not hypothesized (this would be a more intricate and confirmatory analysis). As a consequence, an uncorrected alpha level of 0.050 was used in the analyses. 

## 3. Results

### 3.1. Demographic and Biopsychosocial Characteristics of the Participants: Comparison between Completers and Non-Completers

The demographic and biopsychosocial characteristics of the whole sample are presented in Table 2 and Table 3. There were no significant differences in demographic and biopsychosocial characteristics between completers and non-completers, which supports the generalizability of the study results. Our sample (completers; n = 101) was composed of primiparous Spanish perinatal women between 16 and 36 weeks of gestation (mean = 24.42; SD = 8.62) of approximately 33 years of age, who were in a stable relationship and were well-educated. Most women had some degree of ambivalence toward pregnancy. Three out of four had minimal depressive symptoms, 24% had mild depressive symptoms, 4% had moderate depressive symptoms, and no participants had severe depressive symptoms.

In the postpartum period, 82.2%, 14.8% and 3% of completers presented minimal, mild, and moderate depressive symptoms, respectively. No participant presented severe depressive symptoms in the postpartum.

### 3.2. Cross-Sectional and Longitudinal Bivariate Associations between Pregnancy Biopsychosocial Factors and Pregnancy and Postpartum Depressive Symptoms in the Sample of Completers

Table 4 shows Spearman correlations between prenatal psychosocial factors and prenatal and PPD symptoms in the sample of completers (n = 101). Prenatal depressive symptoms were positively associated with concurrent age (*r* = 0.21; *p* = 0.032), affective ambivalence (*r* = 0.38; *p* < 0.001), neuroticism (*r* = 0.34; *p* < 0.001), and negative affect (*r* = 0.36; *p* = 0.004). Conversely, prenatal depressive symptoms were negatively linked to positive affect (*r* = −0.49; *p* < 0.001). PPD symptoms were related to prenatal age (*r* = 0.27; *p* = 0.006), affective ambivalence (*r* = 0.20; *p* = 0.042), positive affect (*r* = −0.21; *p* = 0.033) and depressive symptoms (*r* = 0.47; *p* < 0.001). Being older, experimenting with more affective ambivalence, reporting less positive affect, and presenting depressive symptoms during pregnancy were associated with increased postpartum depressive symptoms. These correlations were between weak and moderate in strength. Social support and extraversion were not associated with concurrent and prospective depressive symptoms.

As observed in Table 4, there were some significant associations between the biopsychosocial variables included in the study. Neuroticism was positively related to affective ambivalence (*r* = 0.26; *p* = 0.009) and negative affect (*r* = 0.49; *p* < 0.001), as well as negatively linked to extraversion (*r* = −0.21; *p* = 0.038), social support (*r* = −0.26; *p* = 0.010), and positive affect (*r* = −0.38; *p* < 0.001). Positive affect was positively associated to extraversion (*r* = 0.28; *p* = 0.004) and social support (*r* = 0.23; *p* < 0.001). Extraversion correlated positively with social support (*r* = 0.23; *p* = 0.022). Finally, affective ambivalence had a positive relationship with negative affect (*r* = 0.29; *p* = 0.003).

### 3.3. Structural Equation Model Predicting Postpartum Depressive Symptoms from Prenatal Biopsychosocial Factors in the Sample of Completers

**Figure 2:**
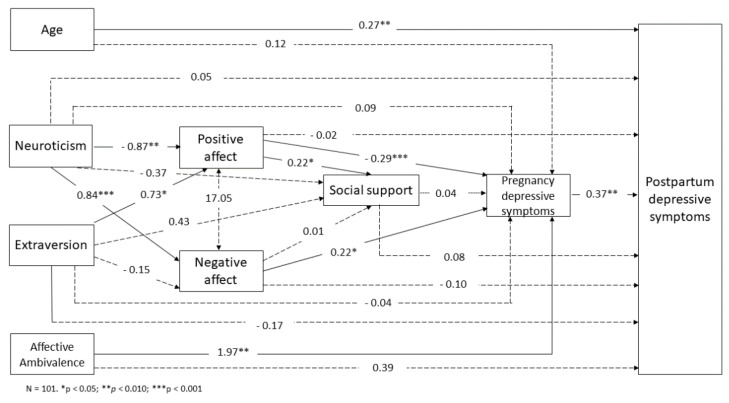
Structural Equation Model predicting depressive symptoms in the sample of completers.

In the model, psychosocial variables were placed according to their expected proximity with PPD (Figure 2). For instance, personality is, theoretically, the most distal factor related to PPD, while pregnancy depressive symptoms would be the most proximal factor to it. 

As shown in Figure 2, distal psychological factors (i.e., neuroticism) were associated with more proximal psychological constructs (i.e., positive and negative affect). Specifically, neuroticism positively contributed to negative affect (*β* = 0.84; *p* < 0.001) and was negatively associated with positive affect (*β* = −0.87; *p* = 0.003). Contrary to neuroticism, extraversion contributed to more positive affect (*β* = 0.73; *p* = 0.018). In an intermediate level, positive affect was associated with more social support at pregnancy (*β* = 0.22; *p* = 0.041). 

A number of cross-sectional associations with prenatal depressive symptoms also emerged. Specifically, positive associations were revealed for affective ambivalence (*β* = 1.97; *p* = 0.003) and negative affect (*β* = 0.22; *p* = 0.024), while positive affect was inversely linked to depressive symptoms (*β* = −0.29; *p* < 0.001). When exploring the longitudinal, prospective associations between study variables and PPD, only age (*β* = 0.27; *p* = 0.010) and prenatal depressive symptoms (*β* = 0.37; *p* = 0.002) predicted PPD. The proposed model showed an excellent fit (χ^2^= 5.072, *p* = 0.535; degrees of freedom = 6; RMSEA < 0.001; 90% RMSEA CI < 0.001–0.118; CFI = 1.000; TLI = 1.045).

## 4. Discussion

The present study aimed at investigating a relatively comprehensive set of biopsychosocial factors associated with perinatal depressive symptoms. Similar to previous research, the biopsychosocial factors included in the study (i.e., age, ambivalence and personality) were cross-sectionally linked to depressive symptoms during pregnancy [8,9,39], and prenatal depressive symptoms were the best predictors of PPD [9,13]. New to this investigation, we observed that the predictive ability of prenatal biopsychosocial factors when predicting PPD becomes negligible, with the exception of age, when prenatal depressive symptoms were accounted for.

### 4.1. Factors Cross-Sectionally Associated with Prenatal Depressive Symptoms

As hypothesized, age, affective ambivalence, neuroticism and negative affect appear to be risk factors for concurrent depressive symptoms, while positive affect is likely to be a protective factor for concurrent prenatal depressive symptoms. A surprising finding was that extraversion and social support did not correlate with concurrent and PPD. 

The relationship between age and perinatal depressive symptoms is not well established in the literature [8]. Our results are consistent with studies suggesting that older women are at higher risk of pregnancy depressive symptoms [40]. However, the strength of the cross-sectional associations found in the present investigation was weak, so the results should be interpreted with caution and replication should be encouraged.

Previous research has supported the association between neuroticism, which is defined as a tendency to experience negative affect when facing a stressor [41], and depressive symptoms [11]. These findings are important because pregnancy is a period in which important challenges are likely to occur due to the changes in the body and the environment [42], so women scoring high in neuroticism are likely to be at higher risk for presenting perinatal depressive symptoms. A similar finding was obtained with negative affect, a personality characteristic closely related to neuroticism. These results are consistent with past research in the perinatal literature [12], and again suggest that there are personality profiles associated with an increased risk of emotional distress in this population that should be taken into consideration in prevention and treatment programs.

In addition to these two risk psychological factors for depressive symptoms, this study investigated the role of two arguably protective factors for depressive symptoms, namely, extraversion and positive affect. Consistent with our expectations, positive affect was inversely associated with the severity of depressive symptoms, which is consistent with the idea that this personality characteristic is a protective factor against depressive symptoms in perinatal women [12]. Surprisingly, though, extraversion, which has been linked to decreased emotional distress in past research [11], and social support [43,44] were not significantly associated with depressive symptoms in our study. It is possible that the role of social support is more evident in disadvantaged populations. Our sample is not likely to be representative of such populations, as participants were generally well-educated adults that reported being in a stable relationship. In the light of the present investigation’s findings, intrapersonal processes (i.e., experienced emotions) appear to be more important for prenatal depressive symptoms than interpersonal elements (i.e., social interactions and support). Another explanation could be that structural social support as measured in the present study (i.e., family support, friends support, and support from others) is negligible compared with functional social support (i.e., instrumental, emotional, informational, etc.). Because the literature in this regard is still scarce, the study results will require replication in similar (i.e., well-educated and maritally stable women) and different populations (i.e., less educated or single women for whom social support can play a more important role). Additionally, it would also be advisable to include different domains of social support (e.g., instrumental and emotional support). As recently suggested, social support to perinatal women should be provided by the right person, delivered at the right time, and should be of the right kind [45].

Overall, the aforementioned cross-sectional findings revealed in the bivariate analyses were supported by SEM, with the exceptions of age and neuroticism. As we predicted, when all biopsychosocial factors were included in a single model, a number of them ceased to significantly contribute to concurrent depressive symptoms, arguably due to shared variance, as indicated by the moderate bivariate associations between psychological variables included in the study. These findings are important as they might guide interventions and research in a more effective way (i.e., reducing the number of target variables due to redundancies and selecting the most robust predictors of outcomes). There is evidence to suggest that neuroticism and negative affect share important variance [46], so the burden of assessment and treatment might be reduced by selecting one of the factors only for research and treatment practices (i.e., negative affect according to the present study findings).

### 4.2. Factors Predicting Postpartum Depressive Symptoms

Consistent with past research [47], our bivariate analyses revealed that the association between psychosocial factors and depressive symptoms decreased with time. Affective ambivalence and positive affect did contribute to depressive symptoms prospectively, but only when investigated in a bivariate manner. 

There is previous evidence to suggest that affective ambivalence is associated with perinatal depressive symptoms [10]. In a context wherein women are forced to view pregnancy as a positive process [11], negative emotions appear to have no or little place. However, as evidenced by the large number of perinatal women who presented some degree of ambivalence in the present investigation (around 70% of respondents), the reality is quite different. Therefore, normalizing and destigmatizing ambivalence toward motherhood should be not only therapeutic goals, but societal aims, so that false negatives are minimized. 

Positive affect was also longitudinally associated with prospective depressive symptoms. There is evidence to suggest that the promotion of positive emotions in perinatal women can be achieved via their participation in pleasant activities [48] and, consistent with the present study findings, this appears to reduce PPD symptoms [49]. Therefore, psychological treatments that encourage the induction of positive emotions or the inclusion of positive-induction modules in existent interventions should be recommended in perinatal women presenting low positive affect.

An interesting longitudinal finding was that only our biological factor, namely age, significantly contributed to PPD above and beyond prenatal depressive symptoms and the remaining psychosocial factors. This is a novel finding which might be attributable to a number of factors that are age-related. For instance, it is possible that older women had unrealistic expectations about motherhood, more difficulties in combining job or house-care duties with maternity, or more risk factor for obstetric and perinatal complications in both the women and the children [50,51], which might lead to increased postpartum depressive symptoms. Because providing information about the changes that occur in the perinatal period appears to be a good intervention for perinatal women [52], adjusting the expectations of older women, anticipating the challenges associated with pregnancy and motherhood, and providing them with abilities to deal with this new situation might be of special interest for older women. Research has shown that women face different stressors during the perinatal period depending on their parity status (i.e., primiparous or multiparous) [53]. Overall, multiparous women appear to experience higher concerns about their lack of social support, while primiparous are more worried about negative body changes or the maternal role [54]. Therefore, the information that should be provided to them and the adjustment of their expectations during pregnancy should consider the woman’s parity status.

Our results showed that, when all biopsychosocial factors are included in a single model, only age and prenatal depressive symptoms were significant predictors of PPD. As anticipated and in line with previous research [8,9], prenatal depressive symptoms were the best predictors of PPD. What these findings suggest is that the intensity of depressive symptoms might remain relatively stable across time [17], so early detection and management should be a priority. Women regularly attend visits with the midwives during the perinatal period, so these appointments would represent good opportunities for mental health assessment and referral to a specialized mental health service when necessary.

Two of the strengths of the present investigation were the implementation of a longitudinal design and the use of technology for assessment. Current clinical practice guidelines recommended longitudinal assessments to detect and identify women at risk for PPD [6]. However, the high costs associated with extensive longitudinal evaluations make the implementation of such screening programs in the current public health systems difficult. While there is some promising evidence on the utility of ICT in this field to overcome dissemination- and cost-related barriers [55], there are research gaps in programs of these characteristics, as the technologies used tend to be old-fashioned (i.e., SMS texts or phone calls) and little research has been conducted in the prenatal period [56]. The use of app-based or web-based screening and treatment methods should be encouraged in this population to maximize the benefits associated with the use of technology, especially during the prenatal period to ensure an early detection and treatment of this problem.

### 4.3. Limitations

The present study certainly has limitations. In our sample, the women were Spanish, well-educated, and predominantly were in a stable couple, so our results may not be generalized to all pregnant women populations. Sample size and dropout rates were also a problem. In this study, the participants were initially 266 perinatal women, but the longitudinal nature of the investigation and the number of included measures likely influence negatively the response rates (i.e., some women responding to a part of the assessment only) and dropouts. In fact, one of the present study goals was to explore whether communalities between constructs exist. This represents important information for reducing assessment protocols to a more manageable set. Another solution to long assessments is the validation of single-item measures against full-length traditional scales, which appears to be feasible and important when using technology (i.e., apps) for repeated assessment [57]. Gamification or gift cards are additional methods to increase adherence to longitudinal assessments. It is important to note that several measures exist to assess depressive symptoms during the perinatal period. Some authors found that the BDI-II, the measure used in the present study, could overestimate the prevalence of depressive symptoms in this population due to its extensive assessment of somatic symptoms [58]. Somatic symptoms are indeed prevalent and underestimated during the perinatal period, which explains why some authors recommended their assessment [59]. Given that both the Edinburgh Postnatal Depression Scale (EPDS) and the BDI-II seem to be accurate in evaluating depressive symptoms [60], future studies should explore whether the prevalence of symptoms in perinatal women is inflated when the BDI-II is administered. An additional limitation in the study was the focus on self-report measures. While this is a frequent practice and provides important information about the subjective state of the individual, the fidelity of the data (i.e., honesty, bias, or understanding and interpretation of items) cannot be guaranteed. This is important when assessing sensitive information, such as ambivalence. However, it is also possible that the assessment procedure used (i.e., online as opposed to face-to-face) actually promoted a sense of anonymity and, thus, enhanced honesty. Another potential problem in the study refers to the use of post hoc questions. The majority of questionnaires administered were full-length scales validated in Spanish populations. However, an important construct, namely pregnancy ambivalence, was measured using an ad hoc item. This practice might represent a threat to the comparability of results. Finally, it is important to note that, despite the fact that a set of important biopsychosocial factors associated with perinatal depressive symptoms was selected, the list is far from complete. For example, biological factors, such as reproductive and stress hormones, psychological variables, such as beliefs and perceived anxiety control, social factors such as marital satisfaction and peer support, and contextual information (i.e., seasonal time frame or seasonal light exposure), have also been linked with depressive symptomatology in perinatal research [15,61], but were not included in the present study for feasibility reasons.

## 5. Conclusions

The assessment of a set of biopsychosocial factors in the present study provides a broader picture of the relationship between biological (age), psychological (affective ambivalence and personality characteristics), and social (social support) factors, and concurrent and prospective depressive symptoms in perinatal women. Our results confirm the need to repeatedly evaluate depressive symptoms in perinatal women. Our SEM analyses revealed that (a) prenatal depressive symptoms were the best biopsychosocial predictors of PPD, (b) for adult women (>18 years old), being younger appears to be a protective factor against perinatal depressive symptoms, especially in the postpartum period, (c) ambivalence about pregnancy and negative affect are related to increased concurrent but not prospective depressive symptoms, and (d) positive affect is associated with reduced concurrent depressive symptoms. The study findings suggest that biopsychosocial longitudinal assessments and intervention protocols that begin during pregnancy and continue in the postpartum are required for this population. In the light of our results, such treatment programs should emphasize the importance of emotional regulation strategies to increase positive affect and to reduce negative affect in women. A recent example is the Unified Protocol for the transdiagnostic treatment of emotional disorders [62,63], but it also includes educational components to help adjust the women’s expectations and face the challenges associated with motherhood, especially in older women. From this study and past experience, we recommend the use of ICT in such programs (i.e., blinded treatments combining onsite and online treatment and ecological momentary assessment with apps) to minimize some of the dissemination and stigma barriers of traditional assessments methods. 

## Figures and Tables

**Table 1:** Inclusion and exclusion criteria to participate in the study.

Inclusion	Exclusion
Being pregnant (weeks 16 to 36)	Not being able to read and answer questions in Spanish
Over 18 years of age	
Having internet access	
Signing the informed consent form	

**Table 2:** Biopsychosocial variables during pregnancy and postpartum.

Variable	Non-Completers		Completers		Comparison	
	Mean (SD; Range)	N	Mean (SD; Range)	N	*U*	*p*
Age	32.59 (4.39; 18–42)	165	33.54 (3.88; 23–42)	101	7474.00	0.157
Affective Ambivalence	0.93 (0.73; 0–3)	165	0.86 (0.74; 0–3)	101		
No	26.1%	43	30.7%	31	7869.00	0.390
Yes	73.9%	122	69.3%	70		
Neuroticism	3.77(3.43; 0–12)	165	3.69 (2.99; 0–12)	101	8191.00	0.815
Extraversion	8.10 (2.93; 0–12)	165	8.54 (2.67; 1–12)	101	7678.00	0.279
Positive Affect	29.70 (9.78; 0–50)	139	29.68 (8.92; 0–50)	101	6735.00	0.592
Negative Affect	16.50 (7.00; 0–40)	139	15.88 (5.82; 0–32)	101	6754.40	0.617
Social Support	75.13 (10.92; 12–84)	123	76.97 (7.84; 42–82)	101	5927.00	0.551
Pregnancy Depressive Symptoms	11.37 (7.40; 2–33)	134	10.21 (5.49; 1–26)	101	6499.50	0.603
Minimal	73.9%	99	72.3%	73	3446.00	0.602
Mild	11.2%	15	23.7%	24	169.50	0.765
Moderate	9.7%	13	4%	4	21.00	0.563
Severe	5.2%	7		0		
Postpartum Depressive Symptoms			8.54 (5.53; 0–25)	101		
Minimal			82.2%	83		
Mild			14.8%	15		
Moderate			3%	3		
Severe				0		

**Table 3:** Sociodemographic variables of the sample.

Variable	Non-Completers	Completers	Comparison	
	Frequency (%)	Frequency (%)	χ^2^	*p*
Nationality				
Spanish	154 (93.3)	94 (93.1)	0.07	0.934
Other	11 (6.7)	7 (6.9)		
Educational Level				
<12 years	23 (13.9)	15 (14.9)	0.04	0.837
>12 years	142 (86.1)	86 (85.1)		
Parity				
Primiparous	122 (73.9)	79 (78.2)	0.62	0.429
Multiparous	43 (26.1)	22 (21.8)		
Relationship Status				
Not in a Relationship	42 (25.5)	17 (16.8)	2.70	0.100
In a Relationship	123 (74.5)	84 (83.2)		

Note: sample size was 165 for non-completers and 101 for completers.

**Table 4:** Bivariate correlations between psychosocial variables and depressive symptoms in the sample of completers.

Variable	Age	AM	N	E	PA	NA	SS	PRE Dep	POST Dep
Age	-	0.14	0.17	−0.8	−0.15	0.13	−0.18	0.21 *	0.27 **
AM		-	0.26 **	−0.01	−0.13	0.29 **	−0.17	0.38 ***	0.20 *
N			-	−0.21 *	−0.38 ***	0.49 ***	−0.26 **	0.34 ***	0.16
E				-	0.28 **	−0.05	0.23 *	−0.15	−0.18
PA					-	−0.17	0.34 ***	−0.49 ***	−0.21 *
NA						-	−0.03	0.36 ***	0.10
SS							-	−0.16	−0.10
PRE Dep								-	0.47 ***
POST Dep									-

Note: AM, affective ambivalence; N, neuroticism; E, extraversion; PA, positive affect; NA, negative affect; SS, social support; PRE dep, pregnancy depressive symptoms; POST dep, postpartum depressive symptoms. *** *p* < 0.001; ** *p* <0.010; * *p* < 0.050. Sample size is 101.
